# Health-related quality of life of adult post COVID-19 condition patients three years after infection and patient characteristics associated with change over time: a longitudinal analysis from the CORFU study

**DOI:** 10.1007/s11136-025-04090-y

**Published:** 2025-10-17

**Authors:** Marcela M. Suazo Guevara, Sophie F. Waardenburg, Dorthe O. Klein, Gouke J. Bonsel, Erwin Birnie, Marieke S. J. N. Wintjens, Bas C. T. van Bussel, Susanne van Santen, Chahinda Ghossein-Doha, Michiel C. Warlé, Lotte M. C. Jacobs, Bena Hemmen, Bas L. J. H. Kietselaer, Gwyneth Jansen, Stella C. M. Heemskerk, Juanita A. Haagsma, Sander M. J. van Kuijk

**Affiliations:** 1 Department of Clinical Epidemiology and Medical Technology Assessment, Maastricht University Medical Center+, Maastricht, The Netherlands; 2 Department of Anesthesiology and Pain Medicine, Maastricht University Medical Center+, Maastricht, The Netherlands; 3 EuroQol Group Executive Office, Rotterdam, The Netherlands; 4 Department of Intensive Care Medicine, Maastricht University Medical Center+, Maastricht, The Netherlands; 5 Cardiovascular Research Institute Maastricht (CARIM), Maastricht University, Maastricht, The Netherlands; 6 Care and Public Health Research Institute (CAPHRI), Maastricht University, Maastricht, The Netherlands; 7 Department of Cardiology, Maastricht University Medical Center+, Maastricht, The Netherlands; 8 Department of Cardiology, Erasmus University Medical Center Rotterdam, Rotterdam, The Netherlands; 9 Department of Surgery, Radboud University Medical Center Nijmegen, Nijmegen, The Netherlands; 10 Adelante Centre of Expertise in Rehabilitation and Audiology, Hoensbroek, The Netherlands; 11 Department of Cardiovascular Disease, Mayo Clinic, Rochester, MN USA; 12 Department of Cardiology, Zuyderland Medical Center, Heerlen, The Netherlands; 13 Department of Obstetrics and Gynaecology, Maastricht University Medical Center, Maastricht, The Netherlands; 14 GROW School for Oncology and Reproduction, Maastricht University, Maastricht, the Netherlands; 15 Department of Public Health, Erasmus University Medical Center Rotterdam, Rotterdam, The Netherlands

**Keywords:** HRQoL, Post COVID-19 condition, Changes in quality of life, COVID-19, EQ-5D-5L

## Abstract

**Purpose:**

Post COVID-19 Condition (PCC) negatively impacts Health Related Quality of Life (HRQoL) and can persist for years after initial infection. This study examined long term HRQoL of COVID-19 survivors with PCC to explore whether and how HRQoL changes over time, and to determine which patient characteristics are associated with changes.

**Methods:**

COVID-19 survivors from the Dutch CORona Follow-Up (CORFU) study were prospectively followed for up to 3 years post-infection using self-report questionnaires. PCC was defined on presence of symptoms reported at the 2-year time point. Within-person change in EQ-5D-5L utility and EQ VAS scores were analyzed from the 2-year to the 3-year time point. Changes were described using summary statistics, and associations with patient characteristics were analyzed using multivariable linear regressions.

**Results:**

Among 158 participants that met PCC definition 2-years after infection, mean utility and EQ VAS change score was 0.01 (95%CI − 0.02,0.04), and 0.9 (95%CI − 1.6,3.4), respectively. Based on EQ utility change scores, 30% experienced a decrease, 32% an increase, and 37% no change. Similar distributions were observed for EQ VAS. Female sex was associated with decreasing utility scores. Increasing utility scores were associated with female sex of older age when compared to younger ones, and with people that initially reported social participation problems..

**Conclusion:**

Individual changes in HRQoL within a year vary widely in the PCC population. Our findings suggest that certain subgroups of individuals with PCC are more vulnerable to prolonged declines in HRQoL.

**Supplementary Information:**

The online version contains supplementary material available at 10.1007/s11136-025-04090-y.

## Abstract (plain language summary)

Some people continue to have symptoms long after their COVID-19 infection. This condition is called post COVID-19 condition (PCC). It affects people in different ways, making it hard to measure and compare how much it impacts their health. One way to do this is by asking people directly how they feel their health has been affected, this is called health-related quality of life (HRQoL). People with PCC often report worse quality of life, more difficulty returning to work, and problems in their social life compared to those without PCC. We wanted to know whether people’s quality of life keeps getting worse over time, improves, or stays the same—and whether this depends on personal or clinical characteristics. We followed a group of people in the Netherlands who had COVID-19 and who had PCC two years after infection. We looked at how their HRQoL changed from year 2 to year 3. We found that people’s experiences differ: some reported better health, some worse, and some stayed the same. People with more existing health problems and women were more likely to get worse. Older women were more likely to improve than younger ones. Overall, there is no single pattern of recovery for PCC. Some people may be more at risk of long-term problems and may need more attention and support over time.

**Supplementary Information:**

The online version contains supplementary material available at 10.1007/s11136-025-04090-y.

## Introduction

Post COVID-19 condition (PCC) refers to the presence of one or more symptoms after the acute phase of a probable or confirmed SARS-CoV-2 infection, that cannot be explained by an alternative diagnosis [1, 2]. The origin of the symptoms ranges across cardiopulmonary, circulatory, cognitive, neurological and immunological domains, among others [3–5]. Once present, PCC can persist for several years. Studies show that 55–70% of people still have symptoms two years after infection, and around 50% have them even after three years [6–9].

PCC negatively impacts health related quality of life, and contributes to functional and mental health impairment, interferes with work and daily life activities, and impairs social engagement [6, 7, 10–16]. It also imposes an additional layer of health burden, worsening pre-existing health conditions and altogether increasing healthcare needs [17]. PCC not only causes an initial decline in HRQoL, but its persistent symptoms can lead to long-term or progressive disabilities that further compromise HRQoL over time [18–22]. PCC symptom trajectories have shown to differ by e.g. age, sex, and the presence of comorbidity [23, 24], thus progression of HRQoL may vary between patients. Populations initially vulnerable to COVID-19, such as the elderly, persons with preexisting disabilities or (multi)comorbidity, racial or ethnic minority groups, and those with lower socioeconomic status [25–28], may face additional disadvantages if they develop PCC, as it can lead to even greater health and social consequences. Women of young age have shown to be a specific subgroup with higher risk of PCC, and more severe symptoms [11, 29–32], and thus, could also be more susceptible to PCC consequences.

As seen in some diseases, progression or recovery depends on an interaction of biological, psychological and social factors [33, 34]. In the case of PCC, predicting individual prognosis, both in terms of symptoms persistence and HRQoL progression, is challenging. At this stage no agreed effective medical or social strategy exists to improve outcomes in general or specific subgroup [35, 36]. Although changes in self-reported HRQoL require cautious interpretation, they may still reflect aspects of disease progression in PCC especially when considering individual patient characteristics. However, with most evidence on HRQoL on PCC patients only available for early periods after initial infection, there is limited understanding of how HRQoL evolves over time in later stages of PCC. Additionally, to what extent the course of HRQoL at later stages of the disease vary by patient characteristics remains unclear. Therefore, the aim of this study was to examine the HRQoL of COVID-19 survivors with PCC across time, to explore whether and how HRQoL changes over time, and to determine which patient characteristics are associated with differences in these changes.

## Methods

### Study design

This a longitudinal study part of the CORona Follow-Up study (CORFU). The CORFU study combined multiple Dutch COVID-19 cohort studies and a national survey assessed in the general Dutch population [37]. Cohort-specific measurements of the acute phase of COVID-19 were complemented by follow-up questionnaires sent at several time points and up to two years. CORFU was registered at ClinicalTrials.gov (NCT05240742), ethical approval was obtained from the medical research ethics committee azM/UM (METC2021-2990) and from the local committees of the participating cohorts. The subsequent Long CORFU study added a three-year follow questionnaire to CORFU’s data collection. It received ethical approval from the medical research ethics committee azM/UM (METC2021-2990-A-2) and from the local committees of the participating cohorts. In both studies, participants provided informed consent prior to receiving the questionnaire, tailored to the initiating cohort study in which they participated. This longitudinal study was conducted from the two- to the three-year follow-up moments, as this period had the greatest availability of data for repeated measures. Two and three years follow up data were collected between March 2023 and February 2024.

### Study population

We used a subpopulation of the CORFU study cohorts, consisting of participants of Bernhoven Early Detection of Vascular Damage after COVID-19 (COVAS) [38]; Maastricht Intensive Care COVID cohort (MaastrICCht) [39]; and ZuydErLand COVID-19 regiStry (ELVIS) [40]). The MaastrICCht cohort included patients admitted to the Intensive Care Unit (ICU). Both the COVAS and ELVIS cohorts included patients admitted to the ward or (also) to the ICU, and patients admitted at the emergency room but who were subsequently sent home for recovery. All participants had a confirmed infection of SARS-CoV-2 during March-December of 2020. SARS-CoV-2 infection was assessed by a positive polymerase chain reaction (PCR) or a positive scored computed tomography of the chest (score 4 or 5 on COVID-19 reporting and data system (CO-RADS)). Participants were at least 18 years of age at the moment of cohort admission and had sufficient knowledge of the Dutch language. For the current study, the additional inclusion criteria was having participated in both the two and three year follow-up measurements, and meeting the PCC definition.

### Data collection and measurement

Data was collected using self-report questionnaires sent digitally or by paper to all cohort participants at both follow-up moments. The questionnaires included clinical characteristics (severity of acute disease, pre-existing health conditions) and sociodemographic characteristics (age, sex, ethnicity) at time of initial infection, and current education, living arrangement, employment status and severity of problems related to social participation.

It also included questions on the severity of a list of general symptoms at the moment of completing the questionnaire, rated on a five-level Likert scale, where “1” represents the absence of the symptom, and “5” represents extreme problems. The symptoms that were assessed were problems with cognition, fatigue, sleep problems, loss of appetite, loss of smell and taste, cough, problems with breathing, pain when breathing, chest pain and discomfort, palpitations, dizziness, swollen ankles/feet and muscle weakness/soreness. Followed by questions on the onset and severity of symptoms relative to the first SARs-CoV-2 infection.

The questionnaire also included the EQ-5D-5L instrument, which assesses five health dimensions: mobility, self-care, usual activities, pain/discomfort, and anxiety/depression, using a five-level Likert scale. Likert scale, where “1” represents the absence of problems, and “5” represents extreme problems. From the dimension scores, a single utility score can be calculated using validated value sets for each country’s population. Utility scores can range from less than 0 (where 0 is the value of a health state equivalent to being dead; negative values representing states valued as worse than death) to 1 (the value of full health)[41]. The EQ-5D-5L also includes a Visual Analogue Scale (EQ VAS), in which the participants rate their current health from 0 (worst imaginable health) to 100 (best imaginable health).

### Definition of variables

We determined the presence of PCC at the 2-year follow-up moment and defined it as having at least one symptom with a score 3 or higher, and the symptom was either not present before the SARS-CoV-2 infection, or, if present before infection, it had worsened. The patient characteristics that were evaluated were sociodemographic, clinical and social. Sociodemographic characteristics included sex, age and ethnicity, and, others at the time of the two-year follow-up, including working status, living arrangement, and level of education. Level of education was aggregated according to the highest level attained, using the International Standard Classification of Education (ISCED) 2011 and Eurostat aggregates: ISCED levels 1–4 were grouped as ‘Low/Medium’ and levels 5–8 as ‘High’ [42]. Clinical characteristics included severity of acute COVID-19, which was determined by the treatment setting of acute disease (ICU, hospital ward, home), and the number of other pre-existing health conditions (e.g. asthma, diabetes). The number of pre-existing health conditions was grouped as none, one, or more than one. Social characteristics included problems related to social participation (i.e. doing things together with others: education, church attendance, cultural) at two year follow-up, which was dichotomized as “No problems” if scored 1 or 2, and “Having problems” if 3–5 in the Likert scale.

### Statistical methods

We used summary statistics to describe patients’ sociodemographic and clinical characteristics. Missing data was quantified as count and percentage. To assess any possible bias due to attrition, we compared characteristics of the participants lost to follow-up in the period between 2 and 3 years and the participants of the 3-year follow-up.

We then applied stochastic regression imputation to all variables to avoid loss of power by the exclusion of incomplete records. We calculated EQ-5D-5L index scores using the Dutch value set [43]. Two and three-year follow-up EQ-5D-5L dimension frequency was described using count and percentages, and Median (Q1, Q3) utility scores and EQ VAS were calculated. We excluded ethnicity from further analyses due to low counts of the non-Dutch group.

To analyze changes in repeated measurements of HRQoL, we first computed absolute differences for the EQ-5D utility and EQ VAS scores of each participant. We defined the Utility change score as: EQ utility at 3 year follow-up (post)—EQ utility at 2 year follow-up (pre) and EQ VAS change score as: EQ VAS at 3 year follow-up(post)—EQ VAS at 2 year follow-up (pre). For interpretation, a threshold of + / − 0.06 was selected to account for measurement error in EQ utility change scores and of + / − 4.0 for EQ VAS [44]. Due to EQ utility and EQ VAS scale characteristics (higher scores indicate better health) direction of changes are interpreted as follows: A negative change represents a deterioration of health in terms of HRQOL indicated further as ‘decline’, and a positive change represents a health gain in terms of HRQOL, indicated further as ‘improvement’. Summary statistics and box plots were used to describe and visualize change scores distribution. The one-sample T-test was used to test whether mean change scores were significantly different from zero. The distribution of participants with each direction of changes was summarized using percentages, and for each direction the change scores summarized with median (min, max). We performed additional stratified analysis by initial levels of HRQoL (at two year follow-up), to explore the distribution of direction of change scores across levels. We classified the initial levels of HRQoL as low, moderate and high; for the EQ-5D-5L utility we used cutoff points < 0.5, >  = 0.50 and < 0.8, and 0.8–1.0, and for the EQ VAS we used 1–60, >  = 60 and < 80, and 80–100, respectively. Alluvial plots were used to illustrate the distribution of participants from each subgroup across the observed directions of change scores.

We then tested associations between patient characteristics and change scores. First, we tested associations individually using linear regressions and then we included all characteristics in one multivariable linear regression model. A positive regression coefficient indicated that a group experienced on average a (greater) increase (or a lower decrease) of HRQoL over time compared to the reference group. In contrast, a negative coefficient indicated the group experienced, on average, (less) increase ( or greater decrease) of HRQoL over time compared to the reference group. For the final interpretation of the coefficient, we used the direction of the average change scores (unadjusted) for each group. Additionally, we tested the hypothesis that the subgroup of young women is more vulnerable to HRQoL deterioration over time. For this, we also tested the interaction between age and sex in the multivariable model and interpreted the coefficients.

To assess whether the magnitude and direction of the associations found in the main regression analyses were influenced by initial HRQoL, we performed a sensitivity analysis by repeating the linear regression models, stratified by initial HRQoL levels (low, moderate, and high). For all regression analyses, (un)adjusted regression coefficients (betas), and 95% confidence intervals (95% CI) were reported. Statistical significance was defined as p-values less than 0.05. R version 4.4.1 was used for data analysis.

## Results

### Description of participants and HRQoL outcomes

**Fig. 1:**
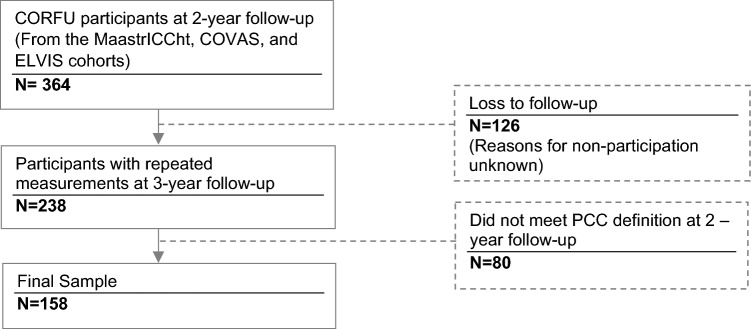
Flow diagram of inclusion of participants to final sample

A total of 158 participants met inclusion criteria (Fig. 1). Most participants were male (68%), the mean (SD) age was 64 (12) and most identified as ethnically Dutch (97%). More than half of the participants (66%) reported having at least one pre-existing health condition. Most participants were admitted to the hospital ward (64%) or to the ICU (27%) during acute COVID-19 (Table 1), and 135 (85%) still had PCC at the 3-year follow-up. Compared to those loss-to- follow-up, participants of the 3-year follow-up were less frequently female (39% vs 27%). However, no significant differences were found in the proportion of individuals with PCC, or in the mean HRQoL at the 2-year follow-up (Online Resource 1). Non-participation reasons were unknown. The most frequent PCC-related symptoms at 2-year follow-up were fatigue, muscle pain/weakness, and problems with sleeping (Online Resource 2). Missing data frequency for EQ-5D-5L dimensions was < 2%. Following imputation, the median (Q_1_, Q_3_) EQ-5D utility at 2 and 3-years follow-up was 0.739 (0.596, 0.821) and 0.743 (0.592, 0.817), respectively, and the median (Q_1_, Q_3_) EQ VAS score for both moments was 64.0 (50.0, 75.0) and 64.0 (50.3,73.8). Distribution of severity of problems for each of the EQ-5D-5L dimensions is available in Online Resource 3.

**Table 1:** Characteristics of study participants

Characteristic	Missing^1^	N = 158^2^
Sex	0 (0%)	
Male		107 (68%)
Female		51 (32%)
Age (at inclusion)	0 (0%)	64 (12)
Ethnicity	1 (0.6%)	
Dutch		152 (97%)
Other		5 (3.2%)
Level of education	3 (1.9%)	
High		36 (23%)
Low/medium		119 (77%)
Working status	12 (7.6%)	
Employed		35 (24%)
Household/Caretaker		1 (0.7%)
Partially due to health		16 (11%)
Retired		72 (49%)
Sick leave, incapacity, unemployed		22 (15%)
Living arrangement	1 (0.6%)	
Alone		28 (18%)
Only with children, parents or other		6 (3.8%)
Partner, with or without children		123 (78%)
Number of pre-existing health conditions	0 (0%)	
None		53 (34%)
One		49 (31%)
> 1		56 (35%)
Social participation	10 (6.3%)	
No problems		119 (80%)
Having problems		29 (20%)
Severity of acute COVID-19 illness	0 (0%)	
Home		15 (9.5%)
Hospital Ward		101 (64%)
ICU		42 (27%)

^1^ N Missing (% Missing)

^2^ n (%); Mean (SD)

^*^Sex, age, ethnicity, number of preexisting health conditions and severity of acute COVID-19 illness are at the time of the initial acute disease. Level of education, working status, living arrangement, problems with social participation are at 2-year follow-up

### Within-person changes in HRQoL outcomes

**Fig. 2:**
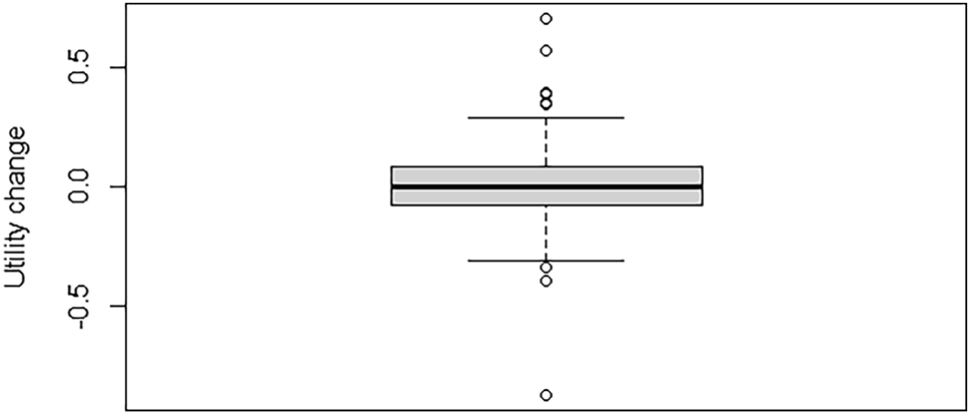
Box plot of the distribution of EQ utility change scores

**Fig. 3:**
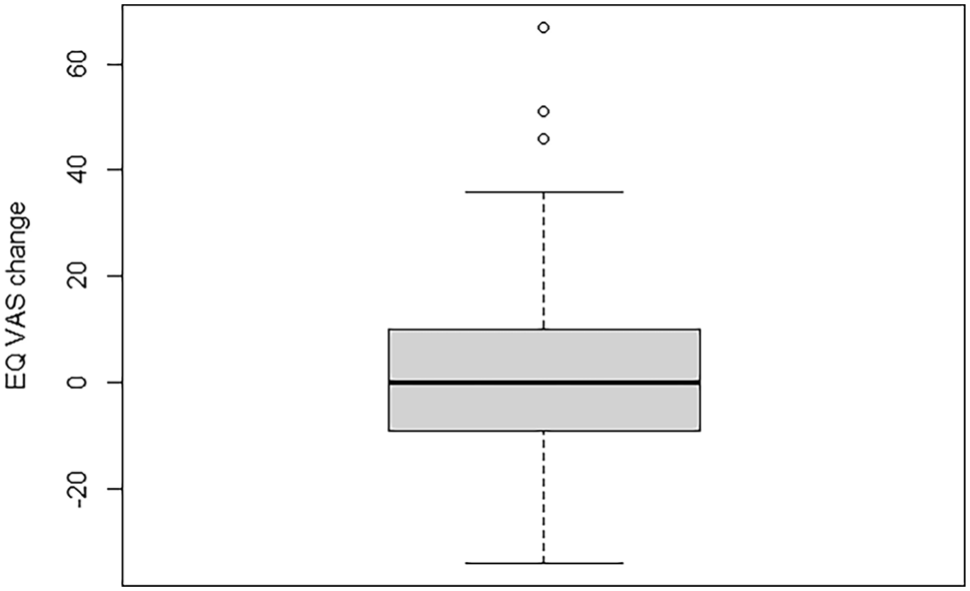
Box plot of the distribution of EQ VAS change scores

**Fig. 4:**
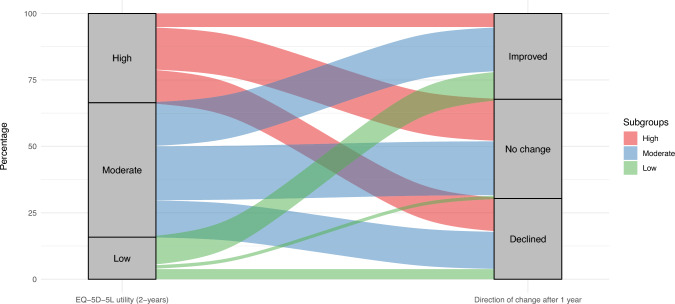
Alluvial plot of the distribution of participants across the observed directions of change in EQ utility scores

**Fig. 5:**
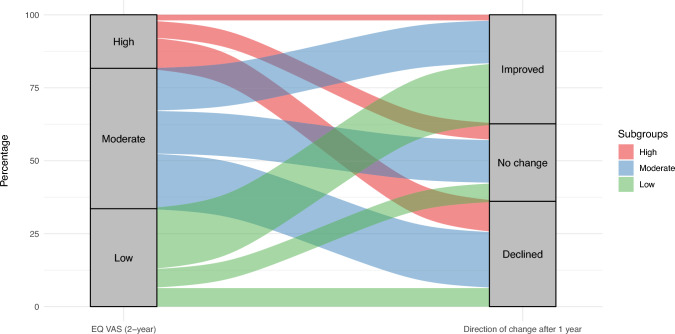
Alluvial plot of the distribution of participants across the observed directions of change in EQ VAS scores

Mean change scores showed no significant difference in both the utility or EQ VAS scores. The mean within-person change in utility score was 0.008 (95% CI − 0.02 to 0.04, *p* = 0.553), and the mean change in EQ VAS was 0.9 (95% CI − 1.6 to 3.4, *p* = 0.467). As shown in Figs. 2 and 3, magnitude of EQ utility and EQ VAS change scores varied widely in both positive and negative directions. The overall distribution of direction of change and change scores (expressed median (Min, Max)) was: 30% showed a decline (− 0.12 (− 0.875, − 0.066)), 32% an improvement (0.157 (0.061, 0.703)), and 37% no change (0.0 (− 0.06, 0.06)) in utility. For EQ VAS scores, the distribution was similar, with 36% experiencing a decline (− 10 (− 5, − 34)), 37% an improvement (14 (5,67)), and 27% no change (0 (− 4, 4). If participants had low initial EQ utility, the predominant tendency was improvement. In contrast, within those with high utility, no change was more common. For the whole sample, No change of EQ utility was most common if participants initially were (already) healthy (high baseline EQ utility). These distributions can be further visualized in Figs. 4 and 5 and described in Online Resource 4.

### Patient characteristics associated with change scores

Multivariable regression analysis showed that mean utility change scores differed significantly by sex. After adjusting for other factors, female sex had 0.10 units lower mean change scores in utility compared to male. EQ utility change scores did not differ significantly by age. We observed a significant interaction between female sex and age > 67 years (0.14 units of mean change score higher). If compared to younger women, older aged women had on average a positive change. Also, participants reporting having problem with social participation had higher mean utility (0.09) and EQ VAS (11) change scores compared to those without problems (Tables 2 and 3).

**Table 2:** Regression analysis on EQ Utility change scores by patient sociodemographic and clinical characteristics

Characteristic	Unadjusted				Adjusted		
	Mean (SD) change score	Beta	95% CI^*1*^	*p* value	Beta	95% CI^*1*^	*p* value
Sex							
Male	0.02 (0.17)	–	–		–	–	
Female	− 0.02 (0.19)	− 0.04	− 0.10, 0.02	0.167	− 0.10	− 0.18, − 0.02	0.012
Age group							
< 67	0.03 (0.20)	–	–		–	–	
>= 67	− 0.02 (0.14)	− 0.05	− 0.10, 0.01	0.102	− 0.05	− 0.14, 0.04	0.239
Working status							
Employed	− 0.01 (0.11)	–	–		–	–	
Household/Caretaker	0.16 (NA)	0.17	− 0.18, 0.52	0.339	0.19	− 0.18, 0.57	0.310
Retired	− 0.01 (0.19)	− 0.01	− 0.07, 0.06	0.846	0.00	− 0.09, 0.09	0.975
Sick leave, incapacity, unemployed	0.08 (0.24)	0.08	− 0.01, 0.17	0.068	0.07	− 0.03, 0.17	0.188
Working partially due to health	0.05 (0.19)	0.06	− 0.04, 0.16	0.240	0.03	− 0.08, 0.13	0.647
Level of education							
High	0.00 (0.14)	–	–		–	–	
Low/medium	0.01 (0.19)	0.01	− 0.06, 0.07	0.870	0.01	− 0.07, 0.08	0.875
Living arrangement							
Alone	0.00 (0.16)	–	–		–	–	
Only with children, parents or other	0.00 (0.17)	− 0.01	− 0.16, 0.15	0.949	0.02	− 0.15, 0.18	0.815
Partner, with or without children	0.01 (0.18)	0.01	− 0.07, 0.08	0.814	0.00	− 0.08, 0.08	0.972
Severity of initial disease							
Home	− 0.04 (0.18)	–	–		–	–	
Hospital Ward	0.01 (0.17)	0.04	− 0.06, 0.14	0.399	0.03	− 0.08, 0.14	0.554
ICU	0.03 (0.20)	0.07	− 0.04, 0.17	0.215	0.06	− 0.06, 0.17	0.316
Number of pre-existing health conditions							
None	0.02 (0.18)	–	–		–	–	
One	0.01 (0.18)	− 0.01	− 0.08, 0.06	0.719	− 0.01	− 0.08, 0.06	0.797
More than one	0.00 (0.17)	− 0.03	− 0.09, 0.04	0.466	− 0.03	− 0.10, 0.04	0.436
Social participation							
No problems	− 0.01 (0.15)	–	–		–	–	
Having problems	0.11 (0.26)	0.12	0.05, 0.19	< 0.001	0.09	0.00, 0.17	0.043
Sex * Age group							
Female * > = 67					0.14	0.02, 0.27	0.028

^1^ CI = Confidence Interval

*Sex, age, number of pre-existing health conditions and severity of acute COVID-19 illness are at the time of the initial acute disease. Level of education, working status, living arrangement, problems with social participation are at 2-year follow-up

**Table 3:** Regression analysis on EQ VAS change scores by patient sociodemographic and clinical characteristics

Characteristic	Unadjusted				Adjusted		
	Change score mean (SD)	Beta	95% CI^*1*^	*p* value	Beta	95% CI^*1*^	*p* value
Sex							
Male	1.67 (15.84)	–	–		–	–	
Female	− 0.71 (15.10)	− 2.4	− 7.6, 2.9	0.372	− 2.2	− 9.4, 4.9	0.537
Age group							
< 67	2.49 (16.11)	–	–		–	–	
> = 67	− 1.25 (14.71)	− 3.7	− 8.7, 1.2	0.136	0.30	− 7.8, 8.4	0.941
Working status							
Employed	2.02 (16.59)	–	–		–	–	
Household/Caretaker	3.00 (NA)	0.98	− 30, 32	0.951	− 12	− 46, 21	0.467
Retired	− 0.97 (13.87)	− 3.0	− 8.8, 2.8	0.312	− 2.3	− 10, 5.5	0.568
Sick leave, incapacity, unemployed	2.68 (15.47)	0.66	− 7.4, 8.7	0.871	− 2.5	− 12, 6.7	0.595
Working partially due to health	3.69 (20.70)	1.7	− 7.3, 11	0.715	− 4.3	− 14, 5.4	0.386
Level of education							
High	2.36 (14.17)	–	–		–	–	
Low/medium	0.48 (16.02)	− 1.9	− 7.7, 4.0	0.526	− 1.4	− 8.1, 5.3	0.675
Living arrangement							
Alone	− 4.50 (17.50)	–	–		–	–	
Only with children, parents or other	3.50 (10.19)	8.0	− 5.8, 22	0.253	7.8	− 6.9, 23	0.295
Partner, with or without children	2.00 (15.20)	6.5	0.10, 13	0.047	4.8	− 2.2, 12	0.178
Severity of initial disease							
Home	4.20 (10.25)	–	–		–	–	
Hospital Ward	0.56 (17.24)	− 3.6	− 12, 4.9	0.403	− 3.5	− 13, 6.1	0.473
ICU	0.55 (12.91)	− 3.7	− 13, 5.7	0.439	− 3.7	− 14, 6.4	0.470
Number of pre-existing health conditions							
None	1.49 (18.23)	–	–		–	–	
One	2.27 (14.59)	0.77	− 5.3, 6.9	0.803	1.3	− 4.9, 7.5	0.680
More than one	− 0.84 (13.76)	− 2.3	− 8.3, 3.6	0.438	− 1.3	− 7.6, 4.9	0.673
Social participation							
No problems	− 0.93 (13.69)	–	–		–	–	
Having problems	9.07 (20.57)	10	3.8, 16	0.002	11	3.9, 19	0.003
Sex * Age group							
Female * > = 67					0.02	− 11, 11	0.997

^1^ CI = Confidence Interval

^*^Sex, age, number of pre-existing health conditions conditions and severity of acute COVID-19 illness are at the time of the initial acute disease. Level of education, working status, living arrangement, problems with social participation are at 2-year follow-up

### Sensitivity analysis- stratification by HRQoL at 2 years

Stratified analyses showed similar results to the analysis on the total cohort, with some exceptions. The mean difference between female and male sex in utility change scores was higher in those with low utility (− 0.33, 95%CI − 0.67, 0.02), similar in those with moderate utility (− 0.13, 95%CI − 0.25, − 0.01) and reversed sign in those with high utility (0.02, 95%C − 0.07, 0.10), compared to the female effect in the total cohort.The effect size of the interaction between sex and age was also higher for participants with low utility (0.56, 95%CI − 0.10, 1.2), similar in those with moderate utility (0.15, 95%CI − 0.02, 0.32) and lower for those with high utility (0.04, 95%CI − 0.10, 0.19). The effect of having social participation problems was of opposite sign in the low (− 0.04, 95%CI − 0.32, 0.23) and high (− 0.25, 95%CI − 0.46, − 0.04) utility groups (Online Resource 5, 6, and 7).In addition to the patterns consistent to findings of the main analyses, we observed differences between those “Working partially, due to health” compared to those “employed” in the high utility group, however, not all subgroups of Working status were represented.

Online Resource 8, 9, and 10 show the results of the stratified analyses on changes in EQ VAS scores. The association of social participation problems with changes in VAS score were lower in the subgroup with low (2.3, 95%CI − 9.2, 14), and moderate (6.7, 95%CI − 4.1, 17) initial VAS scores. There were no participants with social problems in the high VAS subgroup. Secondary associations were observed for Working partially due to health compared to those Employed in the high VAS group; and also in the subgroup of having multiple preexisting conditions compared to none in the low VAS group.It is important to note that not all sample subgroups were (equally) represented in the stratified HRQoL groups. Online Resources 11 and 12 describe the composition of the social participation groups.

## Discussion

Our study aimed to describe changes in HRQoL between two and three years since COVID-19 infection among individuals reporting PCC symptoms, and to identify patient characteristics associated with these changes. Our findings indicate much between-patient variability in change direction of HRQoL outcomes in the one year period, with both improvements and declines being common. Female sex was associated with declining utility scores, while older age showed higher change scores in the female group. Also, having social participation problems was associated with increasing change scores. Our findings on female sex support prior evidence suggesting that women, particularly younger women, are more vulnerable to long-term impacts of PCC and may experience slower symptom recovery compared to men [29, 46]. Interestingly, these differences were not observed in the EQ VAS change scores. This discrepancy may reflect the limited sensitivity of the EQ VAS to detect changes in health. But it may also reflect sex-related differences in the perception and reporting of health, as suggested by García et al. [47], who noted that women often report lower perceived health due to a complex interaction of social, psychological, and cultural influences. These findings also highlight the importance of further investigating sex-specific factors that may shape both actual health trajectories and subjective experiences of recovery.

Previous studies examining HRQoL trajectories in PCC and COVID-19 survivors populations have reported mixed findings, though some align with our results. Tanguay et al.[48] measured the EQ-5D-5L index over time and identified three HRQoL trajectories. The most common trajectory was “stable high,” followed by “slight decline” and “rapid decline.” In contrast to their findings, our study revealed an increase of HRQoL for almost one third of the population. Also, in their study the lowest baseline utility was associated with the rapid declining trajectory, whereas an increase of HRQoL outcomes were frequent among individuals with low baseline utility in ours. In both studies, stability was most prevalent among individuals with high baseline utility. While their study also found female sex to be associated with greater HRQoL decline, they additionally identified older age (> 65) and moderate-to-severe acute COVID-19 infection as predictors, which we did not observe in our data.

Schaap et al. [49], who followed COVID-19 survivors over one year and measured physical and mental HRQoL using the Dutch Short Form Health Survey (SF-36), reported an association between age and change. They identified that younger age was associated with lower mental HRQoL. Moreover, they found that patient characteristics associated with HRQoL change differed between physical and mental health domains. Their findings also suggested relative stability of changes in HRQoL over time, with no substantial improvements or declines observed at specific time points. Similarly, our study showed stability in the high baseline groups, and both studies did find an improving tendency rather than all declining tendencies like the study by Tanguay et al. However, the domain-specific changes reported in their study cannot be directly compared to our results, as the EQ-5D-5L index is an aggregate score that does not differentiate between physical and mental domains. These results, however, emphasize that long-term HRQoL is highly individualized, with distinct patterns across health domains, challenging the assumption that baseline factors can predict HRQoL outcomes in PCC.

Servier et al. [23] identified three distinct PCC symptom recovery patterns during the first two years following infection: slowly decreasing (most common), rapidly decreasing, and highly persistent. In our sample, a significant proportion of participants still reported symptoms at the three-year mark. This raises questions about whether recovery progresses after two years or plateaus. Understanding these symptom trajectories is essential for interpreting changes in HRQoL over time. While symptom burden likely affects HRQoL, the relationship between the two may not be necessarily proportional. In our study, a substantial number of participants reported persistent PCC symptoms, yet, approximately 30% experienced improvements in HRQoL over the same period. Suggesting that broader aspects of functioning and well-being, beyond symptom severity, contribute to perceived quality of life. Similarly, Wang et al. [50], reported stable low HRQoL despite improving fatigue scores, highlighting a potential disconnect between symptom resolution and perceived changes in health.

Interestingly, participants who reported difficulties with social participation at year two had higher improvement in HRQoL by year three compared to those without problems. This may reflect psychological adaptation among those facing initial social limitations. Over time, such individuals may develop coping mechanisms that enhance health perception. This supports the idea that HRQoL trajectories are influenced not just by symptoms, but also by adaptation and social context, as Wiertz et al. [36] suggest. However, adaptation alone doesn’t fully explain why those with initially better social health tended to decline in the utility analysis. This asymmetry points toward regression to the mean as a plausible explanation. Moreover, participants with initially better social health exhibited a more stable progress as seen in their (unadjusted) mean change scores. Positive changes of greater magnitude might be limited by the instrument’s scale upper limit, partly explaining why changes of greater magnitude were more likely to be observed in participants with lower social health, who were only represented in the initially moderate and low EQ VAS strata.

### Strengths and limitations

The main strength of this study is the three-year follow-up period, allowing detailed exploration of the long-term PCC effects on HRQoL. To our knowledge, few studies have such extended follow-up data on COVID-19 survivors. The widespread use of the EQ-5D-5L in PCC research strengthens the relevance and applicability of our findings. Loss to follow-up did not vary by baseline HRQoL or PCC symptom presence, reducing risk of selection bias. However, differences by sex and social participation suggest selective mechanisms can’t be ruled out. For instance, participants with lower social participation may have dropped out due to worsening health—or improvement and reintegration—introducing uncertainty about bias direction. Although symptom severity was assessed prospectively, determining whether symptoms predated infection relied on recall, and thus recall bias two years post-infection remains a possibility. Other potential influences, such as unrelated health conditions, life events, or reinfection, were not assessed. Men are overrepresented in our sample due to the sampling methods of the included cohorts; however, by using sex-adjusted modelling we account for this imbalance and reduce its potential impact on between-group association. Some subgroups were underrepresented in stratified analyses, likely due to sample size rather than true subgroup distribution differences. Secondary patterns should therefore be interpreted with caution and considered exploratory. Finally, the observed trend, where high baseline health declined and low baseline health improved, may reflect moderate EQ measurement reliability or regression to the mean. Future studies incorporating clinical data and multimethod approaches could help clarify these findings. Future research should formally assess the reliability, minimal (clinical) important change and responsiveness to change of the EQ-5D-5L measures in the context of PCC, focusing in exploring systematic differences between sexes.

## Conclusions

In conclusion, among adult COVID-19 survivors with PCC of the Dutch population, particularly those who were hospitalized due to SARS-CoV-2, progression of HRQoL within one year period differs substantially between individuals, with a proportion experiencing a decline of HRQoL. Magnitude of within-person change of HRQoL differs for patients with different characteristics. Women, and particularly women of younger age were associated with disadvantageous differences in the progression of HRQoL over time. This study suggests that certain subgroups of individuals with PCC are more vulnerable to prolonged declines in HRQoL. Further research is needed to understand which baseline and long-term post-infection factors contribute to these negative changes.

## Supplementary Information

Below is the link to the electronic supplementary material.

Supplementary file1 (PDF 140 KB)

Supplementary file2 (PDF 185 KB)

Supplementary file3 (DOCX 20 KB)

Supplementary file4 (PDF 275 KB)

Supplementary file5 (PDF 364 KB)

Supplementary file6 (PDF 234 KB)

Supplementary file7 (PDF 249 KB)

Supplementary file8 (PDF 251 KB)

Supplementary file9 (PDF 251 KB)

Supplementary file10 (PDF 245 KB)

Supplementary file11 (DOCX 21 KB)

Supplementary file12 (DOCX 19 KB)
