# Supplementary material for: Health-related quality of life of adult post COVID-19 condition patients three years after infection and patient characteristics associated with change over time: a longitudinal analysis from the CORFU study
Source: Qual Life Res. 2025 Oct 17;34(11):3305–17. doi: 10.1007/s11136-025-04090-y (PMC12681495; doi:10.1007/s11136-025-04090-y)
Supplement: Supplementary file 1 — Supplementary file1 (PDF 140 KB) [file 11136_2025_4090_MOESM1_ESM.pdf]

Article title: Health-related quality of life of adult Post Covid-19 Condition patients three years after infection and patient characteristics associated with change over time: A longitudinal analysis from the CORFU study

Journal name: Quality of Life Research

Author names: Marcela M. Suazo Guevara, Sophie F. Waardenburg, Dorthe O. Klein, Gouke J. Bonsel, Erwin Birnie, Marieke S.J.N Wintjens, Bas C.T. van Bussel, Susanne van Santen, Chahinda Ghossein-Doha, Michiel C. Warlé, Lotte M.C. Jacobs, Bena Hemmen, Bas L.J.H. Kietselaer, Gwyneth Jansen, Stella C.M. Heemskerk, Juanita A. Haagsma, Sander M.J. van Kuijk

Affiliation and e-mail address of the corresponding author: Department of Clinical Epidemiology and Medical Technology Assessment, Maastricht University Medical Center+, Maastricht, The Netherlands.

marcela.suazo.guevara@mumc.nl

Table 1. Characteristics of participants who were Loss to Follow-up and participants of both 2 and 3-year follow-up

**Characteristic**	**Overall**, N = 364[1]	**Loss to follow-up**, N = 126[1]	**Sample**, N = 238[1]	**p-value**[2]
Sex				**0.023**
Female	114 (31%)	49 (39%)	65 (27%)	
Male	250 (69%)	77 (61%)	173 (73%)	
Age at inclusion				0.939
<67	207 (57%)	72 (57%)	135 (57%)	
>= 67	157 (43%)	54 (43%)	103 (43%)	
Ethnicity				0.719
Dutch	355 (98%)	124 (98%)	231 (97%)	
Non-Dutch	8 (2.2%)	2 (1.6%)	6 (2.5%)	
Working status				0.317
Employed	93 (29%)	30 (29%)	63 (29%)	
Household/Caretaker	7 (2.2%)	5 (4.9%)	2 (0.9%)	
Partially due to health	25 (7.9%)	8 (7.8%)	17 (7.9%)	
Retired	156 (49%)	48 (47%)	108 (50%)	
Sick leave, incapacity, unemployed	36 (11%)	12 (12%)	24 (11%)	
Level of education				0.387
High	78 (22%)	24 (19%)	54 (23%)	
Low/Medium	283 (78%)	102 (81%)	181 (77%)	
Living arrangement				0.835
Alone	56 (15%)	21 (17%)	35 (15%)	
Alone, with children	10 (2.8%)	4 (3.2%)	6 (2.5%)	
Parents or other	7 (1.9%)	3 (2.4%)	4 (1.7%)	
Partner, with or without children	289 (80%)	97 (78%)	192 (81%)	
Severity of initial COVID-19 illness				0.207
Home	57 (16%)	25 (20%)	32 (13%)	
Hospital Ward	216 (59%)	74 (59%)	142 (60%)	
ICU	91 (25%)	27 (21%)	64 (27%)	
Social participation problems	39 (12%)	9 (8.4%)	30 (14%)	0.159
Number of pre-existing health conditions				0.223
None	136 (37%)	42 (33%)	94 (39%)	
One	115 (32%)	47 (37%)	68 (29%)	
More than one	113 (31%)	37 (29%)	76 (32%)	
EQ-5D Utility at 2-year Follow-up	0.81 (0.70, 0.89)	0.81 (0.74, 0.89)	0.81 (0.68, 0.89)	0.620
EQ VAS score at 2-year Follow-up	70 (60, 80)	70 (60, 80)	70 (60, 80)	0.392

PCC status at 2-year Follow-up				0.688
Non PCC	125 (34%)	45 (36%)	80 (34%)	
PCC	239 (66%)	81 (64%)	158 (66%)	

[1] n (%); Mean (SD)

[2] Pearson's Chi-squared test; Fisher's exact test; Welch Two Sample t-test

Cell counts may differ due to missing data
