# Supplementary material for: Health-related quality of life of adult post COVID-19 condition patients three years after infection and patient characteristics associated with change over time: a longitudinal analysis from the CORFU study
Source: Qual Life Res. 2025 Oct 17;34(11):3305–17. doi: 10.1007/s11136-025-04090-y (PMC12681495; doi:10.1007/s11136-025-04090-y)
Supplement: Supplementary file 2 — Supplementary file2 (PDF 185 KB) [file 11136_2025_4090_MOESM2_ESM.pdf]

Article title: Health-related quality of life of adult Post Covid-19 Condition patients three years after infection and patient characteristics associated with change over time: A longitudinal analysis from the CORFU study

Journal name: Quality of Life Research

Author names: Marcela M. Suazo Guevara, Sophie F. Waardenburg, Dorthe O. Klein, Gouke J. Bonsel, Erwin Birnie, Marieke S.J.N Wintjens, Bas C.T. van Bussel, Susanne van Santen, Chahinda Ghossein-Doha, Michiel C. Warlé, Lotte M.C. Jacobs, Bena Hemmen, Bas L.J.H. Kietselaer, Gwyneth Jansen, Stella C.M. Heemskerk, Juanita A. Haagsma, Sander M.J. van Kuijk

Affiliation and e-mail address of the corresponding author: Department of Clinical Epidemiology and Medical Technology Assessment, Maastricht University Medical Center+, Maastricht, The Netherlands.

marcela.suazo.guevara@mumc.nl

Table 2. Frequency of Post Covid-19 Condition (PCC) related symptoms at two-year follow-up

**Symptoms**	**Missing**[1]	**N = 158**[2]
Fatigue	7 (4.4%)	84 (56%)
Dizziness	3 (1.9%)	24 (15%)
Muscle pain and weakness	1 (0.6%)	75 (48%)
Cough	0 (0%)	26 (16%)
Breathing/shortness of breath	5 (3.2%)	55 (36%)
Pain when breathing	1 (0.6%)	5 (3.2%)
Chest Pain (angina)	4 (2.5%)	8 (5.2%)
Heart Palpitations	2 (1.3%)	17 (11%)
Cognition	1 (0.6%)	53 (34%)
Loss of Smell and taste	0 (0%)	28 (18%)
Sleeping problems	0 (0%)	60 (38%)
Loss of Appetite	0 (0%)	7 (4.4%)
Swollen Feet and ankles	3 (1.9%)	23 (15%)

[1] N Missing (% Missing)
[2] n (%)
