# Supplementary material for: Health-related quality of life of adult post COVID-19 condition patients three years after infection and patient characteristics associated with change over time: a longitudinal analysis from the CORFU study
Source: Qual Life Res. 2025 Oct 17;34(11):3305–17. doi: 10.1007/s11136-025-04090-y (PMC12681495; doi:10.1007/s11136-025-04090-y)
Supplement: Supplementary file 3 — Supplementary file3 (DOCX 20 KB) [file 11136_2025_4090_MOESM3_ESM.docx]

**Article title:** Health-related quality of life of adult Post Covid-19 Condition patients three years after infection and patient characteristics associated with change over time: A longitudinal analysis from the CORFU study

**Journal name:** Quality of Life Research

**Author names:** Marcela M. Suazo Guevara, Sophie F. Waardenburg, Dorthe O. Klein, Gouke J. Bonsel, Erwin Birnie, Marieke S.J.N Wintjens, Bas C.T. van Bussel, Susanne van Santen, Chahinda Ghossein-Doha, Michiel C. Warlé, Lotte M.C. Jacobs, Bena Hemmen, Bas L.J.H. Kietselaer, Gwyneth Jansen, Stella C.M. Heemskerk, Juanita A. Haagsma, Sander M.J. van Kuijk

**Affiliation and e-mail address of the corresponding author:** Department of Clinical Epidemiology and Medical Technology Assessment, Maastricht University Medical Center+, Maastricht, The Netherlands.

marcela.suazo.guevara@mumc.nl

**Table 3.** Distribution of frequency of severity of problems for the EQ-5D-5L dimensions at 2 and 3-year follow-up

| Dimension | (N=158) | |
| --- | --- | --- |
|  | **Follow-up moment** | |
|  | **2-year** | **3-year** |
| Mobility |  |  |
| *No problems* | 47 (30%) | 43 (27%) |
| *Slight problems* | 33 (21%) | 43 (27%) |
| *Moderate Problems* | 53 (34%) | 49 (31%) |
| *Severe Problems* | 24 (15%) | 23 (15%) |
| *Extreme problems* | 1 (0.6%) | 0 (0%) |
| Self-care |  |  |
| *No problems* | 110 (70%) | 112 (71%) |
| *Slight problems* | 32 (20%) | 30 (19%) |
| *Moderate Problems* | 11 (7.0%) | 11 (7.0%) |
| *Severe Problems* | 1 (0.6%) | 3 (1.9%) |
| *Extreme problems* | 4 (2.5%) | 2 (1.3%) |
| Usual Activities |  |  |
| *No problems* | 31 (20%) | 43 (27%) |
| *Slight problems* | 62 (39%) | 40 (25%) |
| *Moderate Problems* | 47 (30%) | 57 (36%) |
| *Severe Problems* | 16 (10%) | 18 (11%) |
| *Extreme problems* | 2 (1.3%) | 0 (0%) |
| Pain and Discomfort |  |  |
| *No problems* | 35 (22%) | 42 (27%) |
| *Slight problems* | 59 (37%) | 49 (31%) |
| *Moderate Problems* | 56 (35%) | 55 (35%) |
| *Severe Problems* | 6 (3.8%) | 10 (6.3%) |
| *Extreme problems* | 2 (1.3%) | 2 (1.3%) |
| Anxiety and Depression | |  |
| *No problems* | 85 (54%) | 94 (59%) |
| *Slight problems* | 43 (27%) | 34 (22%) |
| *Moderate Problems* | 23 (15%) | 27 (17%) |
| *Severe Problems* | 7 (4.4%) | 3 (1.9%) |
| *Extreme problems* | 0 (0%) | 0 (0%) |
|  | | |
