# Supplementary material for: Health-related quality of life of adult post COVID-19 condition patients three years after infection and patient characteristics associated with change over time: a longitudinal analysis from the CORFU study
Source: Qual Life Res. 2025 Oct 17;34(11):3305–17. doi: 10.1007/s11136-025-04090-y (PMC12681495; doi:10.1007/s11136-025-04090-y)
Supplement: Supplementary file 4 — Supplementary file4 (PDF 275 KB) [file 11136_2025_4090_MOESM4_ESM.pdf]

Article title: Health-related quality of life of adult Post Covid-19 Condition patients three years after infection and patient characteristics associated with change over time: A longitudinal analysis from the CORFU study

Journal name: Quality of Life Research

Author names: Marcela M. Suazo Guevara, Sophie F. Waardenburg, Dorthe O. Klein, Gouke J. Bonsel, Erwin Birnie, Marieke S.J.N Wintjens, Bas C.T. van Bussel, Susanne van Santen, Chahinda Ghossein-Doha, Michiel C. Warlé, Lotte M.C. Jacobs, Bena Hemmen, Bas L.J.H. Kietselaer, Gwyneth Jansen, Stella C.M. Heemskerk, Juanita A. Haagsma, Sander M.J. van Kuijk

Affiliation and e-mail address of the corresponding author: Department of Clinical Epidemiology and Medical Technology Assessment, Maastricht University Medical Center+, Maastricht, The Netherlands.

marcela.suazo.guevara@mumc.nl

Table 4. Direction of changes of change scores by subgroups of EQ scores at 2-year follow-up

	**Direction of change scores after one-year**			**Total**
	**Declined**	**No change**	**Improved**	
Overall	30%	37%	32%	100%
Subgroups of EQ utility at 2-year				
Low (<0.5)	24%	8%	68%	100%
Moderate (0.5-0.8)	28%	40%	33%	100%
High (≥0.8)	38%	47%	15%	100%
Overall	36%	27%	37%	100%
Subgroups of EQ VAS at 2-year				
Low (1-59)	18%	18%	66%	100%
Moderate (60- 79)	40%	30%	30%	100%
High (80-100)	59%	31%	10%	100%
