# Supplementary material for: Health-related quality of life of adult post COVID-19 condition patients three years after infection and patient characteristics associated with change over time: a longitudinal analysis from the CORFU study
Source: Qual Life Res. 2025 Oct 17;34(11):3305–17. doi: 10.1007/s11136-025-04090-y (PMC12681495; doi:10.1007/s11136-025-04090-y)
Supplement: Supplementary file 5 — Supplementary file5 (PDF 364 KB) [file 11136_2025_4090_MOESM5_ESM.pdf]

Article title: Health-related quality of life of adult Post Covid-19 Condition patients three years after infection and patient characteristics associated with change over time: A longitudinal analysis from the CORFU study

Journal name: Quality of Life Research

Author names: Marcela M. Suazo Guevara, Sophie F. Waardenburg, Dorthe O. Klein, Gouke J. Bonsel, Erwin Birnie, Marieke S.J.N Wintjens, Bas C.T. van Bussel, Susanne van Santen, Chahinda Ghossein-Doha, Michiel C. Warlé, Lotte M.C. Jacobs, Bena Hemmen, Bas L.J.H. Kietselaer, Gwyneth Jansen, Stella C.M. Heemskerk, Juanita A. Haagsma, Sander M.J. van Kuijk

Affiliation and e-mail address of the corresponding author: Department of Clinical Epidemiology and Medical Technology Assessment, Maastricht University Medical Center+, Maastricht, The Netherlands.

marcela.suazo.guevara@mumc.nl

Table 5. Regression analysis on changes in EQ utility score- Subgroup with low utility at 2-year follow-up

		Unadjusted			Adjusted		
**Characteristic**	**N**	**Beta**	**95% CI**[1]	**p-value**	**Beta**	**95% CI**[1]	**p-value**
Sex	25						
Male		—	—		—	—	
Female		-0.21	-0.42, 0.01	0.060	-0.33	-0.67, 0.02	0.062
Age group	25						
<67		—	—		—	—	
>= 67		-0.13	-0.37, 0.10	0.254	-0.14	-0.53, 0.25	0.451
Working status	25						
Employed		—	—		—	—	
Retired		0.04	-0.56, 0.63	0.902	0.12	-0.65, 0.88	0.743
Sick leave/ incapacity/ unemployed		0.02	-0.58, 0.62	0.952	0.13	-0.76, 1.0	0.745
Working partially due to health		0.11	-0.52, 0.73	0.725	0.29	-0.59, 1.2	0.480
Level of education	25						
High		—	—		—	—	
Low/Medium		0.01	-0.28, 0.31	0.918	0.13	-0.27, 0.52	0.483
Living arrangement	25						
Alone		—	—		—	—	
Only with children, parents or other		-0.18	-0.95, 0.60	0.643	-0.08	-1.2, 1.0	0.877
Partner, with or without children		-0.25	-0.81, 0.31	0.367	-0.30	-0.93, 0.33	0.315
Severity of Initial Disease	25						
Home		—	—		—	—	
Hospital Ward		0.13	-0.20, 0.46	0.434	0.01	-0.45, 0.47	0.954
ICU		0.28	-0.09, 0.64	0.129	0.12	-0.37, 0.61	0.591
Number of pre-existing health conditions	25						
None		—	—		—	—	
One		-0.21	-0.49, 0.06	0.118	-0.26	-0.69, 0.16	0.197
More than one		-0.36	-0.61, -0.12	0.006	-0.32	-0.70, 0.06	0.092
Social participation	25						
No problems		—	—		—	—	
Having problems		0.10	-0.12, 0.32	0.352	-0.04	-0.32, 0.23	0.736
Sex * Age group							
*Female * >= 67*					0.56	-0.10, 1.2	0.088

[1] CI = Confidence Interval

*Sex, age, number of pre-existing health conditions and severity of acute COVID-19 illness are at the time of the initial acute disease. Level of education, working status, living arrangement, problems with social participation are at 2-year follow-up.
