# Supplementary material for: Health-related quality of life of adult post COVID-19 condition patients three years after infection and patient characteristics associated with change over time: a longitudinal analysis from the CORFU study
Source: Qual Life Res. 2025 Oct 17;34(11):3305–17. doi: 10.1007/s11136-025-04090-y (PMC12681495; doi:10.1007/s11136-025-04090-y)
Supplement: Supplementary file 6 — Supplementary file6 (PDF 234 KB) [file 11136_2025_4090_MOESM6_ESM.pdf]

Article title: Health-related quality of life of adult Post Covid-19 Condition patients three years after infection and patient characteristics associated with change over time: A longitudinal analysis from the CORFU study

Journal name: Quality of Life Research

Author names: Marcela M. Suazo Guevara, Sophie F. Waardenburg, Dorthe O. Klein, Gouke J. Bonsel, Erwin Birnie, Marieke S.J.N Wintjens, Bas C.T. van Bussel, Susanne van Santen, Chahinda Ghossein-Doha, Michiel C. Warlé, Lotte M.C. Jacobs, Bena Hemmen, Bas L.J.H. Kietselaer, Gwyneth Jansen, Stella C.M. Heemskerk, Juanita A. Haagsma, Sander M.J. van Kuijk

Affiliation and e-mail address of the corresponding author: Department of Clinical Epidemiology and Medical Technology Assessment, Maastricht University Medical Center+, Maastricht, The Netherlands.

marcela.suazo.guevara@mumc.nl

Table 6. Regression analysis on EQ utility change scores- Subgroup with moderate EQ utility at 2-year follow-up

Characteristic	Unadjusted				Adjusted		
	N	Beta	95% CI[1]	p-value	Beta	95% CI[1]	p-value
Sex	80						
Male		—	—		—	—	
Female		-0.05	-0.13, 0.03	0.229	-0.13	-0.25, -0.01	0.029
Age group	80						
<67		—	—		—	—	
>= 67		-0.03	-0.10, 0.05	0.451	0.06	-0.09, 0.21	0.415
Working status	80						
Employed		—	—		—	—	
Household/Caretaker		0.16	-0.17, 0.50	0.332	0.34	-0.07, 0.76	0.106
Retired		-0.04	-0.13, 0.05	0.341	-0.10	-0.23, 0.04	0.169
Sick leave, incapacity, unemployed		0.04	-0.07, 0.15	0.487	0.12	-0.02, 0.26	0.090
Working partially due to health		0.04	-0.09, 0.18	0.547	0.07	-0.09, 0.23	0.398
Level of education	80						
High		—	—		—	—	
Low/Medium		-0.03	-0.13, 0.06	0.466	-0.03	-0.14, 0.07	0.513
Living arrangement	80						
Alone		—	—		—	—	
Only with children, parents or other		-0.03	-0.21, 0.15	0.755	0.00	-0.23, 0.22	0.991
Partner, with or without children		0.01	-0.08, 0.09	0.898	0.01	-0.09, 0.11	0.877
Severity of Initial Disease	80						
Home		—	—		—	—	
Hospital Ward		0.00	-0.15, 0.16	0.990	0.03	-0.18, 0.24	0.752
ICU		0.01	-0.15, 0.18	0.867	0.02	-0.20, 0.25	0.837
Number of pre-existing health conditions	80						
None		—	—		—	—	
One		-0.02	-0.11, 0.07	0.649	-0.05	-0.15, 0.04	0.281
More than one		-0.04	-0.13, 0.05	0.361	-0.09	-0.18, 0.01	0.090
Social participation	80						
No problems		—	—		—	—	
Having problems		0.06	-0.04, 0.15	0.270	0.00	-0.13, 0.13	0.970
Sex * Age group							
Female * >= 67					0.15	-0.02, 0.32	0.080

[1] CI = Confidence Interval

*Sex, age, number of pre-existing health conditions and severity of acute COVID-19 illness are at the time of the initial acute disease. Level of education, working status, living arrangement, problems with social participation are at 2-year follow-up.
