# Supplementary material for: Health-related quality of life of adult post COVID-19 condition patients three years after infection and patient characteristics associated with change over time: a longitudinal analysis from the CORFU study
Source: Qual Life Res. 2025 Oct 17;34(11):3305–17. doi: 10.1007/s11136-025-04090-y (PMC12681495; doi:10.1007/s11136-025-04090-y)
Supplement: Supplementary file 7 — Supplementary file7 (PDF 249 KB) [file 11136_2025_4090_MOESM7_ESM.pdf]

Article title: Health-related quality of life of adult Post Covid-19 Condition patients three years after infection and patient characteristics associated with change over time: A longitudinal analysis from the CORFU study

Journal name: Quality of Life Research

Author names: Marcela M. Suazo Guevara, Sophie F. Waardenburg, Dorthe O. Klein, Gouke J. Bonsel, Erwin Birnie, Marieke S.J.N Wintjens, Bas C.T. van Bussel, Susanne van Santen, Chahinda Ghossein-Doha, Michiel C. Warlé, Lotte M.C. Jacobs, Bena Hemmen, Bas L.J.H. Kietselaer, Gwyneth Jansen, Stella C.M. Heemskerk, Juanita A. Haagsma, Sander M.J. van Kuijk

Affiliation and e-mail address of the corresponding author: Department of Clinical Epidemiology and Medical Technology Assessment, Maastricht University Medical Center+, Maastricht, The Netherlands.

marcela.suazo.guevara@mumc.nl

Table 7. Regression analysis on EQ utility change score- Subgroup with high utility at 2-year follow-up

		Unadjusted			Adjusted		
**Characteristic**	**N**	**Beta**	**95% CI[1]**	**p-value**	**Beta**	**95% CI[1]**	**p-value**
Sex	53						
Male		—	—		—	—	
Female		0.04	-0.02, 0.11	0.147	0.02	-0.06, 0.11	0.612
Age group	53						
<67		—	—		—	—	
>= 67		-0.01	-0.07, 0.05	0.751	-0.04	-0.13, 0.06	0.435
Working status	53						
Employed		—	—		—	—	
Retired		-0.02	-0.07, 0.04	0.585	-0.01	-0.11, 0.08	0.752
Working partially due to health		-0.17	-0.29, -0.05	0.008	-0.18	-0.30, -0.05	0.006
Level of education	53						
High		—	—		—	—	
Low		0.01	-0.05, 0.07	0.794	0.00	-0.07, 0.08	0.985
Living arrangement	53						
Alone		—	—		—	—	
Only with children, parents or other		-0.06	-0.29, 0.16	0.578	-0.10	-0.33, 0.13	0.396
Partner, with or without children		-0.02	-0.10, 0.06	0.667	0.01	-0.08, 0.09	0.891
Severity of Initial Disease	53						
Home		—	—		—	—	
Hospital Ward		0.04	-0.04, 0.13	0.328	0.04	-0.06, 0.14	0.451
ICU		0.02	-0.08, 0.12	0.701	-0.01	-0.11, 0.10	0.910
Number of pre-existing health conditions	53						
None		—	—		—	—	
One		0.02	-0.05, 0.09	0.609	0.01	-0.07, 0.08	0.887
More than one		0.06	-0.01, 0.13	0.087	0.05	-0.03, 0.12	0.231
Social participation	53						
No problems		—	—		—	—	
Having problems		-0.23	-0.43, -0.02	0.029	-0.25	-0.46, -0.05	0.018
Sex * Age group							
Female * >= 67					0.04	-0.10, 0.19	0.548

[1] CI = Confidence Interval

*Sex, age, number of pre-existing health conditions and severity of acute COVID-19 illness are at the time of the initial acute disease. Level of education, working status, living arrangement, problems with social participation are at 2-year follow-up.
