# Supplementary material for: Health-related quality of life of adult post COVID-19 condition patients three years after infection and patient characteristics associated with change over time: a longitudinal analysis from the CORFU study
Source: Qual Life Res. 2025 Oct 17;34(11):3305–17. doi: 10.1007/s11136-025-04090-y (PMC12681495; doi:10.1007/s11136-025-04090-y)
Supplement: Supplementary file 8 — Supplementary file8 (PDF 251 KB) [file 11136_2025_4090_MOESM8_ESM.pdf]

Article title: Health-related quality of life of adult Post Covid-19 Condition patients three years after infection and patient characteristics associated with change over time: A longitudinal analysis from the CORFU study

Journal name: Quality of Life Research

Author names: Marcela M. Suazo Guevara, Sophie F. Waardenburg, Dorthe O. Klein, Gouke J. Bonsel, Erwin Birnie, Marieke S.J.N Wintjens, Bas C.T. van Bussel, Susanne van Santen, Chahinda Ghossein-Doha, Michiel C. Warlé, Lotte M.C. Jacobs, Bena Hemmen, Bas L.J.H. Kietselaer, Gwyneth Jansen, Stella C.M. Heemskerk, Juanita A. Haagsma, Sander M.J. van Kuijk

Affiliation and e-mail address of the corresponding author: Department of Clinical Epidemiology and Medical Technology Assessment, Maastricht University Medical Center+, Maastricht, The Netherlands.

marcela.suazo.guevara@mumc.nl

Table 8. Regression analysis on EQ VAS change scores - Subgroup with low EQ VAS at 2-year follow-up

Characteristic		Unadjusted			Adjusted		
	N	Beta	95% CI[1]	p-value	Beta	95% CI[1]	p-value
Sex	53						
Male		—	—		—	—	
Female		-4.3	-15, 6.8	0.438	-11	-24, 2.5	0.106
Age group	53						
<67		—	—		—	—	
>= 67		-1.6	-12, 8.8	0.754	6.5	-11, 24	0.455
Working status	53						
Employed		—	—		—	—	
Retired		-19	-33, -4.7	0.010	-17	-36, 2.3	0.084
Sick leave, incapacity, unemployed		-19	-34, -4.7	0.011	-5.7	-24, 12	0.517
Working partially due to health		-10	-27, 6.5	0.222	-4.5	-23, 14	0.624
Level of education	53						
High		—	—		—	—	
Low/Medium		-2.4	-14, 9.3	0.678	3.3	-8.9, 15	0.590
Living arrangement	53						
Alone		—	—		—	—	
Only with children, parents or other		2.3	-27, 31	0.875	13	-18, 45	0.407
Partner, with or without children		2.9	-12, 18	0.697	3.5	-12, 19	0.650
Severity of Initial Disease	53						
Home		—	—		—	—	
Hospital Ward		4.0	-13, 21	0.636	2.0	-15, 19	0.815
ICU		-4.7	-23, 14	0.610	-11	-30, 7.9	0.244
Number of pre-existing health conditions	53						
None		—	—		—	—	
One		-11	-23, 2.1	0.101	-15	-31, 0.45	0.057
More than one		-15	-27, -3.6	0.011	-18	-32, -4.6	0.010
Social participation	53						
No problems		—	—		—	—	
Having problems		6.6	-3.5, 17	0.196	2.3	-9.2, 14	0.687
Sex * Age group							
Female * >= 67					24	-5.0, 54	0.101

[1] CI = Confidence Interval

*Sex, age, number of pre-existing health conditions and severity of acute COVID-19 are at the time of the initial acute disease. Level of education, working status, living arrangement, problems with social participation are at 2-year follow-up.
