# Supplementary material for: Health-related quality of life of adult post COVID-19 condition patients three years after infection and patient characteristics associated with change over time: a longitudinal analysis from the CORFU study
Source: Qual Life Res. 2025 Oct 17;34(11):3305–17. doi: 10.1007/s11136-025-04090-y (PMC12681495; doi:10.1007/s11136-025-04090-y)
Supplement: Supplementary file 9 — Supplementary file9 (PDF 251 KB) [file 11136_2025_4090_MOESM9_ESM.pdf]

Article title: Health-related quality of life of adult Post Covid-19 Condition patients three years after infection and patient characteristics associated with change over time: A longitudinal analysis from the CORFU study

Journal name: Quality of Life Research

Author names: Marcela M. Suazo Guevara, Sophie F. Waardenburg, Dorthe O. Klein, Gouke J. Bonsel, Erwin Birnie, Marieke S.J.N Wintjens, Bas C.T. van Bussel, Susanne van Santen, Chahinda Ghossein-Doha, Michiel C. Warlé, Lotte M.C. Jacobs, Bena Hemmen, Bas L.J.H. Kietselaer, Gwyneth Jansen, Stella C.M. Heemskerk, Juanita A. Haagsma, Sander M.J. van Kuijk

Affiliation and e-mail address of the corresponding author: Department of Clinical Epidemiology and Medical Technology Assessment, Maastricht University Medical Center+, Maastricht, The Netherlands.

marcela.suazo.guevara@mumc.nl

Table 9. Regression analysis on EQ VAS change scores- Subgroup with moderate EQ VAS at 2-year follow-up

		Unadjusted			Adjusted		
**Characteristic**	**N**	**Beta**	**95% CI[1]**	**p-value**	**Beta**	**95% CI[1]**	**p-value**
Sex	76						
Male		—	—		—	—	
Female		-0.04	-6.3, 6.2	0.989	3.1	-7.2, 13	0.552
Age group	76						
<67		—	—		—	—	
>= 67		-3.3	-9.1, 2.6	0.271	-0.38	-11, 9.9	0.940
Working status	76						
Employed		—	—		—	—	
Household/Caretaker		4.4	-22, 31	0.743	-9.0	-42, 24	0.587
Retired		-2.1	-9.1, 4.9	0.549	2.7	-6.7, 12	0.567
Sick leave, incapacity, unemployed		-2.0	-13, 9.3	0.724	1.3	-12, 15	0.848
Working partially due to health		-2.6	-14, 8.8	0.650	-7.3	-20, 5.3	0.253
Level of education	76						
High		—	—		—	—	
Low/Medium		-3.4	-11, 4.7	0.404	-3.6	-14, 6.9	0.492
Living arrangement	76						
Alone		—	—		—	—	
Only with children, parents or other		13	-2.3, 29	0.093	11	-7.8, 31	0.237
Partner, with or without children		8.0	0.95, 15	0.027	7.6	-0.76, 16	0.074
Severity of Initial Disease	76						
Home		—	—		—	—	
Hospital Ward		-7.8	-18, 2.5	0.135	-5.8	-20, 8.2	0.411
ICU		-2.7	-14, 8.3	0.628	-0.81	-15, 13	0.909
Number of pre-existing health conditions	76						
None		—	—		—	—	
One		2.9	-4.2, 10	0.419	1.7	-6.1, 9.6	0.660
More than one		-0.83	-8.0, 6.3	0.818	-2.6	-11, 5.4	0.516
Social participation	76						
No problems		—	—		—	—	
Having problems		2.0	-6.7, 11	0.645	6.7	-4.1, 17	0.220
Sex * Age group							
Female * >= 67					-5.2	-19, 8.8	0.460

[1] CI = Confidence Interval

*Sex, age, number of pre-existing health conditions and severity of acute COVID-19 are at the time of the initial acute disease. Level of education, working status, living arrangement, problems with social participation are at 2-year follow-up.
