# Supplementary material for: Health-related quality of life of adult post COVID-19 condition patients three years after infection and patient characteristics associated with change over time: a longitudinal analysis from the CORFU study
Source: Qual Life Res. 2025 Oct 17;34(11):3305–17. doi: 10.1007/s11136-025-04090-y (PMC12681495; doi:10.1007/s11136-025-04090-y)
Supplement: Supplementary file 10 — Supplementary file10 (PDF 245 KB) [file 11136_2025_4090_MOESM10_ESM.pdf]

Article title: Health-related quality of life of adult Post Covid-19 Condition patients three years after infection and patient characteristics associated with change over time: A longitudinal analysis from the CORFU study

Journal name: Quality of Life Research

Author names: Marcela M. Suazo Guevara, Sophie F. Waardenburg, Dorthe O. Klein, Gouke J. Bonsel, Erwin Birnie, Marieke S.J.N Wintjens, Bas C.T. van Bussel, Susanne van Santen, Chahinda Ghossein-Doha, Michiel C. Warlé, Lotte M.C. Jacobs, Bena Hemmen, Bas L.J.H. Kietselaer, Gwyneth Jansen, Stella C.M. Heemskerk, Juanita A. Haagsma, Sander M.J. van Kuijk

Affiliation and e-mail address of the corresponding author: Department of Clinical Epidemiology and Medical Technology Assessment, Maastricht University Medical Center+, Maastricht, The Netherlands.

marcela.suazo.guevara@mumc.nl

Table 10. Regression analysis on EQ VAS change scores - Subgroup with high EQ VAS at 2-year follow-up

Characteristic	Unadjusted				Adjusted		
	N	Beta	95% CI[1]	p-value	Beta	95% CI[1]	p-value
Sex	29						
Male		—	—		—	—	
Female		1.4	-6.1, 8.8	0.712	-0.49	-12, 11	0.926
Age group	29						
<67		—	—		—	—	
>= 67		-2.6	-10, 5.2	0.499	-3.2	-15, 8.9	0.585
Working status	29						
Employed		—	—		—	—	
Retired		-1.4	-8.0, 5.2	0.664	2.8	-10, 16	0.654
Working partially due to health		-25	-43, -7.4	0.007	-30	-53, -7.5	0.012
Level of education	29						
High		—	—		—	—	
Low/Medium		-2.1	-9.4, 5.3	0.569	-4.5	-14, 4.6	0.311
Living arrangement	29						
Alone		—	—		—	—	
Only with children, parents or other		-5.0	-27, 17	0.641	-5.8	-29, 18	0.605
Partner, with or without children		-0.04	-9.8, 9.8	0.993	0.92	-10, 12	0.866
Severity of Initial Disease	29						
Home		—	—		—	—	
Hospital Ward		-6.1	-18, 5.8	0.301	-1.8	-17, 13	0.800
ICU		-8.3	-22, 5.9	0.241	-7.8	-24, 8.2	0.315
Number of pre-existing health conditions	29						
None		—	—		—	—	
One		4.2	-4.6, 13	0.333	-0.11	-11, 11	0.984
More than one		0.10	-8.7, 8.9	0.981	-4.2	-16, 7.6	0.458
Sex * Age group							
Female * >= 67					-2.1	-21, 17	0.822

[1] CI = Confidence Interval
*Sex, age, number of pre-existing health conditions and severity of acute COVID-19 illness are at the time of the initial acute disease. Level of education, working status, living arrangement, problems with social participation are at 2-year follow-up.
