# Supplementary material for: Health-related quality of life of adult post COVID-19 condition patients three years after infection and patient characteristics associated with change over time: a longitudinal analysis from the CORFU study
Source: Qual Life Res. 2025 Oct 17;34(11):3305–17. doi: 10.1007/s11136-025-04090-y (PMC12681495; doi:10.1007/s11136-025-04090-y)
Supplement: Supplementary file 11 — Supplementary file11 (DOCX 21 KB) [file 11136_2025_4090_MOESM11_ESM.docx]

**Article title:** Health-related quality of life of adult Post Covid-19 Condition patients three years after infection and patient characteristics associated with change over time: A longitudinal analysis from the CORFU study

**Journal name:** Quality of Life Research

**Author names:** Marcela M. Suazo Guevara, Sophie F. Waardenburg, Dorthe O. Klein, Gouke J. Bonsel, Erwin Birnie, Marieke S.J.N Wintjens, Bas C.T. van Bussel, Susanne van Santen, Chahinda Ghossein-Doha, Michiel C. Warlé, Lotte M.C. Jacobs, Bena Hemmen, Bas L.J.H. Kietselaer, Gwyneth Jansen, Stella C.M. Heemskerk, Juanita A. Haagsma, Sander M.J. van Kuijk

**Affiliation and e-mail address of the corresponding author:** Department of Clinical Epidemiology and Medical Technology Assessment, Maastricht University Medical Center+, Maastricht, The Netherlands.

[marcela.suazo.guevara@mumc.nl](mailto:marcela.suazo.guevara@mumc.nl)

**Table 11.** Sociodemographic and clinical characteristics of participants by groups of having and not having social participation problems

| **Characteristic** | **Missing***^1^* | **Overall**, N = 148*^2^* | **Social participation problems** | | **p-value***^3^* |
| --- | --- | --- | --- | --- | --- |
|  |  |  | Not having problems, N = 119*^2^* | Having problems, N = 29*^2^* |  |
| Sex | 0 (0%) |  |  |  | 0.791 |
| Male |  | 99 (67%) | 79 (66%) | 20 (69%) |  |
| Female |  | 49 (33%) | 40 (34%) | 9 (31%) |  |
| Age (at inclusion) | 0 (0%) | 63 (12) | 65 (12) | 58 (11) | **0.009** |
| Level of education | 2 (1.4%) |  |  |  | 0.242 |
| High |  | 33 (23%) | 29 (25%) | 4 (14%) |  |
| Low/Medium |  | 113 (77%) | 89 (75%) | 24 (86%) |  |
| Working status | 3 (2.0%) |  |  |  | **<0.001** |
| Employed |  | 35 (24%) | 33 (28%) | 2 (6.9%) |  |
| Household/Caretaker |  | 1 (0.7%) | 0 (0%) | 1 (3.4%) |  |
| Partially due to health |  | 16 (11%) | 8 (6.9%) | 8 (28%) |  |
| Retired |  | 71 (49%) | 63 (54%) | 8 (28%) |  |
| Sick leave, incapacity, unemployed |  | 22 (15%) | 12 (10%) | 10 (34%) |  |
| Living arrangement | 0 (0%) |  |  |  | 0.249 |
| Alone |  | 27 (18%) | 24 (20%) | 3 (10%) |  |
| Only with children, parents or other |  | 6 (4.1%) | 6 (5.0%) | 0 (0%) |  |
| Partner, with or without children |  | 115 (78%) | 89 (75%) | 26 (90%) |  |
| Number of pre-existing comorbidities | 0 (0%) |  |  |  | 0.863 |
| None |  | 50 (34%) | 39 (33%) | 11 (38%) |  |
| One |  | 45 (30%) | 37 (31%) | 8 (28%) |  |
| >1 |  | 53 (36%) | 43 (36%) | 10 (34%) |  |
| Severity of inital disease | 0 (0%) |  |  |  | >0.999 |
| Home |  | 15 (10%) | 12 (10%) | 3 (10%) |  |
| Hospital Ward |  | 99 (67%) | 79 (66%) | 20 (69%) |  |
| ICU |  | 34 (23%) | 28 (24%) | 6 (21%) |  |
| *^1^* N Missing (% Missing)  *^2^* n (%); Mean (SD)  *^3^* Pearson’s Chi-squared test; Welch Two Sample t-test; Fisher’s exact test  *Sex, age, number of preexisting comorbid conditions and severity of acute COVID-19 illness are at the time of the initial acute disease. Level of education, working status, living arrangement, problems with social participation are at 2-year follow-up.  10 participants were omitted due to missing data in the Social Participation variable | | | | | |
