# Supplementary material for: Health-related quality of life of adult post COVID-19 condition patients three years after infection and patient characteristics associated with change over time: a longitudinal analysis from the CORFU study
Source: Qual Life Res. 2025 Oct 17;34(11):3305–17. doi: 10.1007/s11136-025-04090-y (PMC12681495; doi:10.1007/s11136-025-04090-y)
Supplement: Supplementary file 12 — Supplementary file12 (DOCX 19 KB) [file 11136_2025_4090_MOESM12_ESM.docx]

**Article title:** Health-related quality of life of adult Post Covid-19 Condition patients three years after infection and patient characteristics associated with change over time: A longitudinal analysis from the CORFU study

**Journal name:** Quality of Life Research

**Author names:** Marcela M. Suazo Guevara, Sophie F. Waardenburg, Dorthe O. Klein, Gouke J. Bonsel, Erwin Birnie, Marieke S.J.N Wintjens, Bas C.T. van Bussel, Susanne van Santen, Chahinda Ghossein-Doha, Michiel C. Warlé, Lotte M.C. Jacobs, Bena Hemmen, Bas L.J.H. Kietselaer, Gwyneth Jansen, Stella C.M. Heemskerk, Juanita A. Haagsma, Sander M.J. van Kuijk

**Affiliation and e-mail address of the corresponding author:** Department of Clinical Epidemiology and Medical Technology Assessment, Maastricht University Medical Center+, Maastricht, The Netherlands.

[marcela.suazo.guevara@mumc.nl](mailto:marcela.suazo.guevara@mumc.nl)

**Table 12.** Sociodemographic and clinical characteristics of the subgroup of participants reporting having social participation problems, stratified by sex

| **Characteristic** | **Missing***^1^* | **Overall**, N = 29*^2^* | **Sex** | | **p-value***^3^* |
| --- | --- | --- | --- | --- | --- |
|  |  |  | **Male**, N = 20*^2^* | **Female**, N = 9*^2^* |  |
| Age (at inclusion) | 0 (0%) | 58 (11) | 59 (11) | 57 (11) | 0.710 |
| Level of education | 1 (3.4%) |  |  |  | >0.999 |
| High |  | 4 (14%) | 3 (16%) | 1 (11%) |  |
| Low/Medium |  | 24 (86%) | 16 (84%) | 8 (89%) |  |
| Working status | 0 (0%) |  |  |  | 0.529 |
| Employed |  | 2 (6.9%) | 2 (10%) | 0 (0%) |  |
| Household/Caretaker |  | 1 (3.4%) | 0 (0%) | 1 (11%) |  |
| Partially due to health |  | 8 (28%) | 6 (30%) | 2 (22%) |  |
| Retired |  | 8 (28%) | 6 (30%) | 2 (22%) |  |
| Sick leave, incapacity, unemployed |  | 10 (34%) | 6 (30%) | 4 (44%) |  |
| Living arrangement | 0 (0%) |  |  |  | >0.999 |
| Alone |  | 3 (10%) | 2 (10%) | 1 (11%) |  |
| Only with children, parents or other |  | 0 (0%) | 0 (0%) | 0 (0%) |  |
| Partner, with or without children |  | 26 (90%) | 18 (90%) | 8 (89%) |  |
| Number of pre-existing comorbidities | 0 (0%) |  |  |  | >0.999 |
| None |  | 11 (38%) | 8 (40%) | 3 (33%) |  |
| One |  | 8 (28%) | 5 (25%) | 3 (33%) |  |
| >1 |  | 10 (34%) | 7 (35%) | 3 (33%) |  |
| Severity of initial diseases | 0 (0%) |  |  |  | 0.325 |
| Home |  | 3 (10%) | 1 (5.0%) | 2 (22%) |  |
| Hospital Ward |  | 20 (69%) | 14 (70%) | 6 (67%) |  |
| ICU |  | 6 (21%) | 5 (25%) | 1 (11%) |  |
| *^1^* N Missing (% Missing) | | | | | |
| *^2^* Mean (SD); n (%) | | | | | |
| *^3^* Welch Two Sample t-test; Fisher’s exact test  *Sex, age, number of preexisting comorbid conditions and severity of acute COVID-19 illness are at the time of the initial acute disease. Level of education, working status, living arrangement, problems with social participation are at 2-year follow-up. | | | | | |
